# *Treponema pallidum* Immunohistochemistry is positive in human intestinal Spirochetosis

**DOI:** 10.1186/s13000-017-0676-6

**Published:** 2018-01-22

**Authors:** Rondell P. Graham, Bita V. Naini, Sejal S. Shah, Christina A. Arnold, Rajesh Kannangai, Michael S. Torbenson, Dora M. Lam-Himlin

**Affiliations:** 1 Department of Laboratory Medicine and Pathology, Division of Anatomic Pathology, Mayo Clinic, 200 First Street SW, Rochester, MN 55905 USA; 2 Department of Pathology and Laboratory Medicine, David Geffen School of Medicine, University of California Los Angeles, 10833 Le Conte Ave. Suite 27-061C7 CHS, Los Angeles, CA 90095 USA; 3 Department of Pathology, The Ohio State University Wexner Medical Center, 410 West 10th Avenue, Columbus, OH 43210 USA; 4 Department of Clinical Virology, Christian Medical College, Vellore, 632004 India; 5 Department of Laboratory Medicine and Pathology, Division of Anatomic Pathology, Mayo Clinic, Scottsdale, AZ 85259 USA

**Keywords:** Intestinal spirochetosis, *Treponema*, Diarrhea, *Brachyspira*, Warthin-starry, *B. Aalborgi*, *B. Pilosicoli*

## Abstract

**Background:**

Human intestinal spirochetosis (IS) has been recognized for decades, but whether it represents commensalism or a pathogenic process remains controversial. IS is diagnosed on routine stains with confirmation by silver stains but these stains are labor intensive and slow to read. We evaluated the *Treponema pallidum* immunostain as a diagnostic adjunct for IS.

**Methods:**

We retrieved biopsies from 33 patients with IS for this study. Each case was tested by Warthin-Starry (WS) and *T. pallidum* immunohistochemistry (IHC). Species specific genotyping was performed in 3 cases.

**Results:**

Patients with IS ranged from 22 to 82 years without gender predilection. IS involved normal (*n* = 15), and inflamed (*n* = 5) mucosa and colonic polyps (*n* = 13). Warthin-Starry and *T. pallidum* IHC were positive in all cases including both species of *Brachyspira*. Six (18%) symptomatic patients were treated for IS, and experienced resolution. In patients diagnosed with incidental IS on cancer screening (n = 5), follow up biopsies, without therapy, were negative for IS. *T. pallidum* IHC required 75 min less hands-on time than WS for performance and was faster to interpret.

**Conclusions:**

*T. pallidum* IHC can be used to confirm the diagnosis of IS and is easier to perform and faster to interpret than WS.

Some of these data were presented at the 105th United States and Canadian Academy of Pathology Annual Meeting in Seattle, WA and have been published in abstract form [1].

## Background

Intestinal spirochetosis (IS) is diagnosed on routine sections with identification of a thick band of basophilia at the apical aspect of the intestinal mucosa on low magnification. On high magnification, this basophilic band can be resolved as linearly arranged spiral organisms attached to the apical aspect of the colonocytes at the mucosal surface. The diagnosis is most often confirmed by a silver stain such as Warthin-Starry or the Steiner stain. Although most cases are identified initially by routine hematoxylin and eosin (H&E) stain, the presence of a microvillous brush border along the surface of colonocytes can be a diagnostic pitfall, and confirmatory staining is sometimes necessary.

IS is caused by *Brachyspira* species, *B. aalborgi* and *B. pilosicoli* [2, 3], and has been recognized for decades, but whether it represents commensalism or a pathogenic process is still debated [4–6]. Authors have suggested that IS is often incidentally discovered and has no apparent clinical consequence but in some cases there is a diarrheal illness which resolves after metronidazole treatment and may be associated with an immunocompromised state [5, 7, 8]. It has been suggested that the presence of invasion into the cytoplasm of epithelial cells or lamina propria indicates symptomatic infection, but this has only been examined by ultrastructural study in a total of 5 cases [9, 10]. The *Treponema pallidum* immunostain has been shown to be positive in cases of intestinal spirochetosis in a prior study [8] but this was not the main focus of that study. We therefore evaluated the *Treponema pallidum* immunostain, which cross-reacts with *Brachyspira* species, as a diagnostic tool for IS.

The *T.pallidum* immunostain can be performed by standard automation methods unlike the manual Warthin-Starry method. In addition, the Warthin-Starry stain can be challenging to interpret with significant background staining. As such, we also designed this study to compare the technical hands on time to perform Warthin-Starry and *T. pallidum* immunohistochemistry and the interpretive time for the pathologist reviewing these stains.

## Methods

### Cases

In this institutional review board exempt study, we retrieved colonic biopsies diagnosed as IS from 1998 to 2015 where residual formalin fixed paraffin embedded tissue was available from the authors’ institutional files. The diagnostic slides were reviewed to confirm the diagnosis, assess for concomitant pathology and to select a paraffin block for ancillary study (RPG, BVN, SSS, DLH). We also retrieved 10 control cases (normal colon (*n* = 2), lymphocytic colitis (*n* = 3), cryptosporidiosis (*n* = 4) and active chronic colitis (*n* = 1)). All cases and controls were reviewed and scored after performing special stains (RPG, BVN, DLH).

#### Histochemistry and Immunohistochemistry

Each case was uniformly tested by Warthin-Starry (WS) and *T. pallidum* immunohistochemistry (IHC). For WS, all glassware to be used in stain preparation was rinsed with acidulated water. Twenty and 30 ml solutions of 5% gelatin and 0.15% Hydroquinone were prepared in separate flasks and heated to 60 °C for an hour. Silver nitrate solution (2%) was prepared in a water rinsed flask. A coplin jar and 50 ml graduated cylinder were heated to 60 °C for an hour. The glass slides with formalin fixed paraffin embedded tissue were deparaffinized and then hydrated during that time. Next, the 2% silver nitrate, 5% gelatin and 0.15% hydroquinone were added sequentially to the warmed coplin jar with stirring. This was called the developer solution. The hydrated slides were placed in 1% silver nitrate solution and then into a 43 °C water bath for 30 min. The slides were then transferred to the developer solution and placed in a 43 °C water bath for between 30 s to 1 min with close observation. The slides were then washed quickly and thoroughly in running hot tap water. The slides were then rinsed with distilled water. Each slide was dehydrated in alcohol followed by xylene treatment and coverslipping. IHC for *T. pallidum* was performed as described by Arnold et al. [11] with the exception that the Ventana platform was used instead of the Leica Bond platform.

The hands-on technical time for WS and *T. pallidum* IHC was determined by stopwatch for a group of 11 randomly selected cases simultaneously submitted for staining. Also, the microscope time for these 11 cases and 5 control cases was assessed for two blinded independent observers (MST and SSS) who reviewed each case and control with WS and *T. pallidum* IHC with a 10-day interval between WS and *T. pallidum* IHC review. During the 10-day interval, these observers (MST and SSS) were part of the daily routine clinical service providing a sufficient wash out between interpretation of WS and *T. pallidum*.

#### Genotyping

Species specific genotyping was performed in 3 cases using a previously published method [12]. In brief, genomic DNA was extracted from spirochetes that had been microdissected from formalin-fixed, paraffin-embedded histologic sections and was subjected to polymerase chain reaction (PCR) analysis. PCR primers were designed to amplify a non-conserved region of the bacterial 16S ribosomal RNA gene, permitting identification of *B. aalborgi* and *B. pilosicoli* based on sequence alignment.

#### Clinical information

Patient demographics, abdominal complaints (diarrhea, abdominal pain/cramping, bloating, rectal bleeding), indication for colonoscopy, colonoscopy reports, clinical follow up and follow up biopsies were examined blinded to the results of ancillary tests.

## Results

We identified 33 patients with IS including 18 men and 15 women ranging from 22 to 82 years. The indications for colonoscopy were as follows: colorectal cancer screening (*n* = 19), unexplained diarrhea (*n* = 8), abdominal pain (*n* = 2), hematochezia (n = 2) and anemia or low vitamin B12 (n = 2). Four patients were human immunodeficiency virus (HIV) positive. The colonoscopic impressions were available for 32 (97%) patients and were normal (*n* = 12), polyps (*n* = 13), diverticular disease only (*n* = 2), diverticular disease with polyps (n = 1), aphthous ulceration (n = 2), changes of colitis (n = 1; in a patient with a history of Crohn’s disease), ulcer in rectum but otherwise normal (n = 1).

**Fig. 1:**
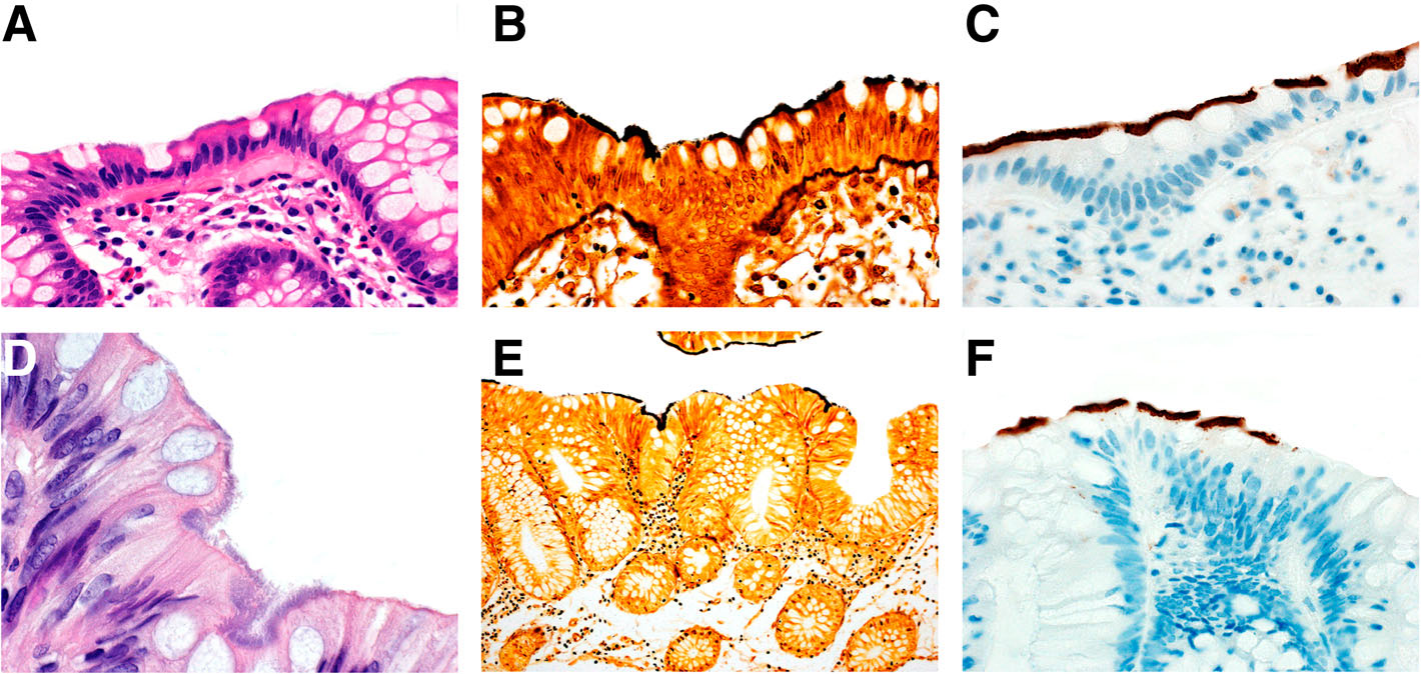
**a** Hematoxylin and eosin stained section of colonic epithelium showing the characteristic histology of intestinal spirochetosis (Original magnification × 600). **b** The Warthin –Starry stain highlights the *Brachyspira* species along the apical aspect of the colonic epithelium (Original magnification × 600). **c** The *T. pallidum* immunostain robustly highlights the *Brachyspira* species without significant background staining (Original magnification × 600). **d** A tubular adenoma removed as part of cancer surveillance screening is colonized by intestinal spirochetosis (Original magnification ×1000). **e** The Warthin-Starry stain decorates the organisms (Original magnification ×400). **f** The *T. pallidum* immunostain is similarly positive for intestinal spirochetosis (Original magnification ×1000)

IS involved normal mucosa (*n* = 15), inflamed mucosa (*n* = 5) and both the neoplastic and non-neoplastic mucosa adjacent to adenomatous (*n* = 8), serrated (*n* = 3) and hyperplastic polyps (n = 2). Representative photomicrographs are shown in Fig. 1.

### *T. pallidum* IHC is 100% sensitive for intestinal spirochetosis and highlights tissue invasion

**Fig. 2:**
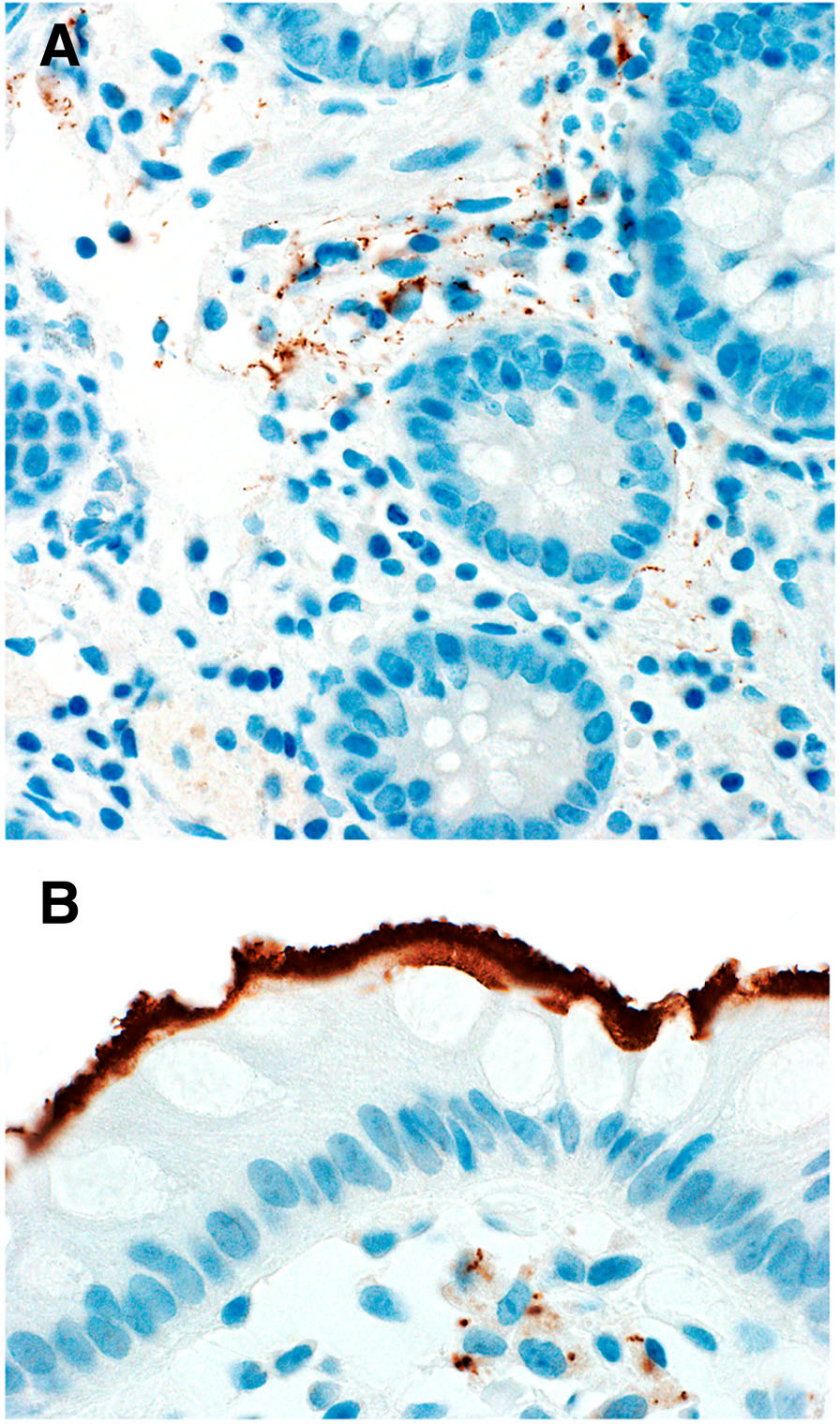
**a** The *T. pallidum* immunostain highlights numerous spiral organisms in the lamina propria. Some macrophages also contain ingested organisms. These findings are unapparent on H&E or Warthin Starry stains (Original magnification ×600). **b** This *T. pallidum* immunostain shows not only the colonization of the surface epithelium characteristic of intestinal spirochetosis, but also reveals lamina propria macrophages harboring spiral organisms (Original magnification ×1000)

WS (*n* = 33 of 33) and *T. pallidum* IHC (*n* = 31 of 31) were positive in all cases of IS (Fig. 1) including both species of *Brachyspira*. *T. pallidum* IHC displayed no background staining. Spirochetes were highlighted within colonic epithelium, lamina propria and lamina propria macrophages by the *T. pallidum* immunostain in 30 (97%) of cases (Fig. 2). Only surface epithelial organisms were seen by WS. None of the 10 controls were positive by WS or *T. pallidum* IHC.

### *T. pallidum* IHC and Warthin-starry detect both B. Aalborgi and B. Pilosicoli

Genotyping results from 3 cases revealed 2 cases of *B. aalborgi* and 1 case of *B. pilosicoli* affecting a male patient with HIV who presented with a history of diarrhea. No clinical follow up was available for these 3 cases but *T.pallidum* IHC and WS were positive in each of the three cases, confirming the sensitivity of these stains to both genotypes of *Brachyspira*.

### Clinical follow up of intestinal Spirochetosis

In 8 patients, follow up biopsies/clinical information were available for review. Four of the patients with clinical follow up were treated with metronidazole because of diarrheal symptoms. Each of the four patients experienced symptom resolution. In one of these patients, symptoms recurred approximately 8 months later and a second course of metronidazole therapy was prescribed with sustained resolution thereafter. The other 3 patients remained symptom free. In 2 of these patients, follow up biopsies were negative for IS. The other patients were not re-biopsied.

Four of the patients with follow up were not treated – each of these patients was undergoing endoscopy for colorectal cancer screening. Biopsies from subsequent screening colonoscopies were reviewed and did not show IS (median 5 year interval). A total of 6 (18%) patients were treated for IS including 2 without clinical follow up.

### *T. pallidum* IHC required less hands-on technical time and was faster to interpret than Warthin-starry

*T. pallidum* IHC required 15 min hands-on technical time compared to 90 min for WS. Two independent pathologists blindly reviewed the hematoxylin and eosin stained sections, *T. pallidum* IHC and WS for the same 16 cases including 11 examples of IS and 5 controls while being timed. In the first sitting, the pathologists were timed reviewing the routine stains and WS and then they were timed 2 weeks later reviewing the routine stains and *T. pallidum* IHC. The 2 pathologists took 10 and 18 min to review the cases with WS and 6 (40% less time) and 12 (33% less time) minutes respectively to review the cases with *T. pallidum* IHC. The average time saved was 5 min over the set of 16 cases or 0.3 min per case.

## Discussion

Human IS is identified infrequently in approximately 1% of colonic biopsies [8, 13]. The diagnosis is made on routine sections and confirmed most often by silver stains such as Warthin-Starry. The prevalence has been reported to be increased in resource limited countries and in immunocompromised hosts [5, 13, 14]. Despite being recognized histologically since the nineteenth century [15], there remain several controversial points regarding IS. First, there continues to be debate as to whether IS is a pathogenic condition or whether it represents commensalism [2, 4–7, 9, 10, 12–14]. Another point of contention is focused on whether IS can involve adenomatous epithelium or not. Some authors have proposed that adenomatous epithelium bears a disrupted brush border which is inhospitable to *Brachyspira* organisms and as such IS does not colonize adenomatous epithelium [16]. Others have shown individual examples of IS involving adenomatous polyps [17]. Yet another controversial issue is the detection of invasion by *Brachyspira* organisms. Existing literature describes detection of Brachyspira organisms from the serum of immunocompromised patients with *B.pilosicoli* [18, 19] but not from patients with *B. aalborgi*. Ultrastructural studies have described invasion into the colonic epithelium [9, 10] but no correlation with symptoms has been examined in a series with both symptomatic and asymptomatic cases.

From our study, *T. pallidum* IHC is positive in 100% of IS, a finding also noted by recent observers [20]. Furthermore, *T. pallidum* IHC is easier to perform and faster to interpret than the commonly used WS stain. This is useful information for laboratories trying to optimize the use of hands-on time for technical staff and for pathologists who are trying to optimize sign out time in time-constrained settings. Other ancillary immunohistochemical, molecular and in situ hybridization assays have been used to detect intestinal spirochetosis. *Brachyspira* PCR is reportedly used in the Netherlands [21]. Tanahashi et al. have described that *T. pallidum* IHC and also *Mycobacterium* IHC highlight the organisms of IS [22]. Jensen et al. described a fluorescent in situ hybridization assay which is specific for *Brachyspira* species [23] and a Swedish group described an immunostain for *Brachyspira* species [24]. Unfortunately, these antibodies are not available in the United States and fluorescent microscopy is not readily available or practical for routine gastrointestinal biopsy examination. These limitations in availability impede the implementation of these assays. In contrast, *T. pallidum* IHC is widely available in the United States and so others can readily employ it for confirmation of the diagnosis of IS. In their description, Willen et al. [24] posit that IHC is preferable to silver stains and our study provides objective evidence to support their hypothesis.

We also were able to highlight IS involving multiple adenomatous polyps and serrated polyps. This is similar to findings from prior case reports ^16^ and confirms that IS can involve neoplastic epithelium. In the authors’ experience, *T. pallidum* IHC can cross-react with luminal bacteria giving rise to faint staining of individual bacilli and cocci separate from the tissue. This should not be interpreted as evidence of IS. Also, as expected, *T. pallidum* IHC shows no reactivity with the microvillous brush border of the colonocytes, and therefore can be used to exclude IS when a prominent brush border is present. Notably, IS is reported to share similar risk factors as syphilis but the morphologies of the 2 diseases are quite distinct. Syphilis is characterized by active colitis with prominent plasma cell infiltration and occasional ulceration in contrast to the above described features of IS.

With regard to the issue of tissue invasion, the *T. pallidum* IHC highlighted organisms within colonic epithelial cells and the lamina propria. Interestingly, rare spiral organisms were visualized engulfed within lamina propria macrophages (Fig. 2). This along with observations from prior ultrastructural studies [9, 10] suggests that *Brachyspira* species are capable of tissue invasion. However, it is not possible to definitively confirm the species of these organisms within the lamina propria in view of the fact that the *T. pallidum* antibody cross reacts with other bacterial organisms. Notably, the frequency of detection of organisms within the lamina propria by IHC is in excess of the frequency of symptoms and there were no symptomatic differences between cases where there was invasion and those without. Taken together, these data indicate need for further study into the capacity of *Brachyspira* organisms to invade and the physiology surrounding symptomatic infection.

Less than half (42%) of patients diagnosed with IS during the study were symptomatic indicating that if IS is a pathogenic infection it is of low virulence as has been suggested by others. There were 4 patients with HIV with IS but 3 of these patients were lost to follow up and in 1 patient, clinical follow up is pending at the time of writing. The lack of follow up precludes assessment of the impact of immunodeficiency on clinical characteristics. Indeed, a limitation of this study is the limited clinical follow up overall. Nonetheless, where follow up information as available, symptomatic patients (i.e. those with diarrheal illnesses) who were treated for IS experienced resolution of symptoms. Interestingly, asymptomatic patients who were followed up experienced resolution without any antimicrobial treatment. Spontaneous resolution in asymptomatic individuals has been described by several others [4, 7, 8, 12, 13]. Taken together, it would appear that antimicrobial treatment is not indicated in incidentally diagnosed IS, but treatment to prevent forward transmission may be prudent.

## Conclusion

In summary, *T. pallidum* IHC is 100% sensitive for the confirmation of the diagnosis of IS and is faster to perform and interpret than WS. As such, *T. pallidum* IHC is a useful adjunct in the diagnosis of IS.

## Abbreviations

H&E: Hematoxylin and eosin
HIV: Human immunodeficiency virus
IHC: Immunohistochemistry
IS: Intestinal spirochetosis
PCR: Polymerase chain reaction
WS: Warthin-Starry
